# Structure refinement of the δ_1*p*_ phase in the Fe–Zn system by single-crystal X-ray diffraction combined with scanning transmission electron microscopy

**DOI:** 10.1107/S2052520613034410

**Published:** 2014-03-17

**Authors:** Norihiko L. Okamoto, Katsushi Tanaka, Akira Yasuhara, Haruyuki Inui

**Affiliations:** a Department of Materials Science and Engineering, Kyoto University, Yoshida, Sakyo-ku, Kyoto 606-8501, Japan; b Center for Elements Strategy Initiative for Structure Materials (ESISM), Kyoto University, Yoshida, Sakyo-ku, Kyoto 606-8501, Japan; c Department of Mechanics, Kobe University, Rokkodai-cho, Nada-ku, Kobe, Hyogo 657-8501, Japan; d EM Application Group, JEOL Ltd, 1-2 Musashino 3-chome, Akishima, Tokyo 196-8558, Japan

**Keywords:** synchrotron radiation, focused ion beam (FIB), intermetallic compound, icosahedron, scanning transmission electron microscopy

## Abstract

The structure of the δ_1*p*_ phase in the iron−zinc system has been refined by single-crystal synchrotron X-ray diffraction combined with scanning transmission electron microscopy. The large hexagonal unit cell of the δ_1*p*_ phase with the space group of *P*6_3_/*mmc* comprises more or less regular (normal) Zn_12_ icosahedra, disordered Zn_12_ icosahedra, Zn_16_ icosioctahedra and dangling Zn atoms that do not constitute any polyhedra. The unit cell contains 52 Fe and 504 Zn atoms so that the compound is expressed with the chemical formula of Fe_13_Zn_126_. All Fe atoms exclusively occupy the centre of normal and disordered icosahedra. Iron-centred normal icosahedra are linked to one another by face- and vertex-sharing forming two types of basal slabs, which are bridged with each other by face-sharing with icosioctahedra, whereas disordered icosahedra with positional disorder at their vertex sites are isolated from other polyhedra. The bonding features in the δ_1*p*_ phase are discussed in comparison with those in the Γ and ζ phases in the iron−zinc system.

## Abstract 2

The structure of the title compound has been refined by single-crystal synchrotron X-ray diffraction combined with spherical-aberration-corrected scanning transmission electron microscopy. The structure consists of iron-centred normal and disordered Zn_12_ icosahedra, zinc-centred Zn_12_ icosahedra, zinc-centred Zn_16_ icosioctahedra, and dangling Zn atoms that do not constitute any polyhedra.

## 1. Introduction   

The iron−zinc binary system has been intensively investigated in both thermodynamic and crystallographic aspects over several decades (Ghoniem & Lohberg, 1972 ▶; Bastin *et al.*, 1974 ▶; Perrot & Dauphin, 1988 ▶; Petersen *et al.*, 1988 ▶; Reumont *et al.*, 2000 ▶; Su *et al.*, 2001 ▶; Nakano *et al.*, 2005 ▶; Kainuma & Ishida, 2005 ▶; Johansson *et al.*, 1968 ▶; Brandon *et al.*, 1974 ▶; Koster & Schoone, 1981 ▶; Brown, 1962 ▶; Hong & Saka, 1997 ▶; Uwakweh *et al.*, 2000 ▶; Belin & Belin, 2000 ▶; Belin *et al.*, 2000 ▶) partly because of the enormous importance of hot dip galvannealed (GA) steels which find wide applications in exposed automobile body panels owing to its high corrosion resistance, weldability and paintability (Marder, 2000 ▶). The coating layer of GA steels usually consists of five intermetallic compounds, Γ (Fe_3_Zn_10_), Γ_1_ (Fe_11_Zn_40_), δ_1*k*_ (FeZn_7_), δ_1*p*_ (FeZn_10_) and ζ (FeZn_13_) phases, which appear in the zinc-rich domain of the iron−zinc binary phase diagram (Ghoniem & Lohberg, 1972 ▶; Kubaschewski, 1982 ▶; Nakano *et al.*, 2005 ▶; Kainuma & Ishida, 2005 ▶). Despite the intensive investigations of the intermetallic phases (Allen & Mackowiak, 1963 ▶; Ghoniem & Lohberg, 1972 ▶; Bastin *et al.*, 1977 ▶), it is only recently that the existence of two distinct phases (δ_1*k*_ and δ_1*p*_) in the δ_1_ region has been accepted (Kainuma & Ishida, 2005 ▶; Hong & Saka, 1997 ▶). The δ_1_ (δ_1*k*_/δ_1*p*_) phase was long considered to be relatively ductile so as to play an important role in the deformation of the coating when GA steels are bent, stretched and drawn during the forming process, because coating failure is mitigated when the coating layer consists largely of the δ_1_ (δ_1*k*_/δ_1*p*_) phase (Kato *et al.*, 1994 ▶). However, we have recently revealed that both of the δ_1*k*_ and δ_1*p*_ phases are extremely brittle exhibiting no plastic deformation in compression tests of polycrystalline micropillar specimens (Okamoto *et al.*, 2013 ▶). The structural information about the δ_1*k*_ and δ_1*p*_ phases is limited, although it is indispensable for understanding their plastic deformability.

Belin *et al.* (Belin & Belin, 2000 ▶) performed crystal structure refinement of the δ_1*p*_ phase by single-crystal X-ray diffraction with a laboratory X-ray source (Mo *K*α), and the results will be described in next section. On the other hand, Hong *et al.* (Hong & Saka, 1997 ▶) found a stark difference in selected-area electron diffraction (SAED) patterns between the δ_1*p*_ and δ_1*k*_ phases. Additional streaks are observed in SAED patterns of the δ_1*k*_ phase at positions indicating that the *a*-axis dimension of the δ_1*k*_ phase is three times that of the δ_1*p*_ phase. Hong *et al.* (Hong & Saka, 1997 ▶) thus concluded that the δ_1*k*_ phase has a superlattice structure based on the δ_1*p*_ phase. However, further refinement of the crystal structure of the δ_1*k*_ phase has yet to be made. We have recently tried to perform crystal structure refinement of the δ_1*k*_ phase (as well as the δ_1*p*_ phase) by scanning transmission electron microscopy (STEM). In the course of our STEM investigation of the δ_1*p*_ phase, however, we have found some evidence that is against the results of the crystal structure refinement of the δ_1*p*_ phase by Belin *et al.* (Belin & Belin, 2000 ▶), which might arise from the difficulties in unambiguously distinguishing iron from zinc with a powerless laboratory X-ray source due to the small difference in the X-ray scattering factors between iron and zinc. We have therefore decided to re-investigate the crystal structure of the δ_1*p*_ phase, based on which superlattice structure of the δ_1*k*_ phase is formed, paying special attention to distinguishing iron from zinc with a synchrotron X-ray source of high luminance and coherence, combining with ultra-high-resolution STEM with a correction of spherical-aberration (Cs) that permits direct observation of individual atomic columns.

## 2. Crystal structure of the δ_1*p*_ phase previously reported   

**Figure 1:**
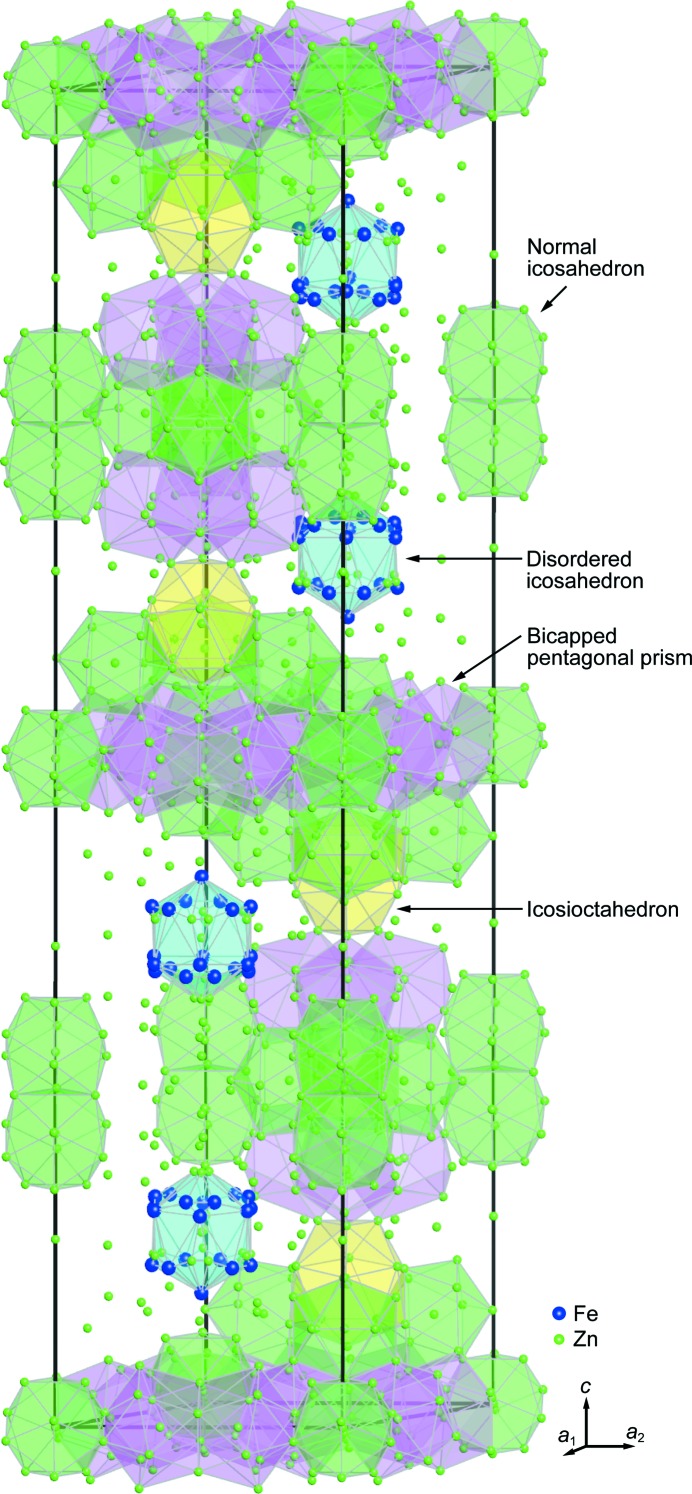
Structural model of the δ_1*p*_ phase reported by Belin *et al.* (Belin & Belin, 2000 ▶).

**Figure 2:**
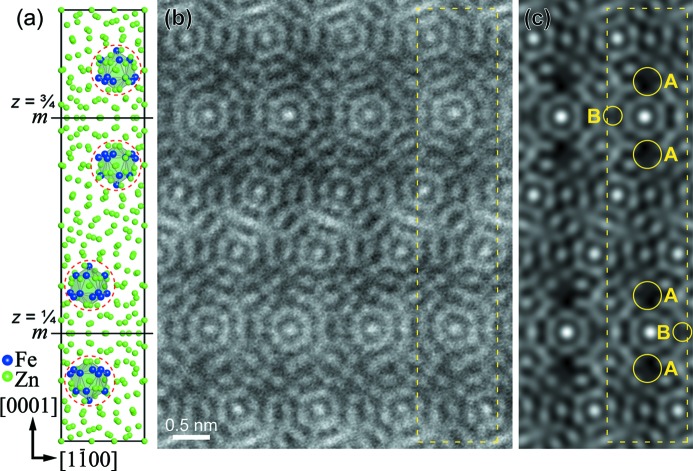
(*a*) [11
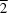
0] projection of the structural model reported by Belin *et al.* (Belin & Belin, 2000 ▶). (*b*) Experimental HAADF image taken along the [11
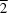
0] zone axis by high-resolution STEM imaging. (*c*) HAADF image calculated based on the structural model depicted in (*a*). The framed area in (*b*) and (*c*) corresponds to the projection of the unit cell.

Belin *et al.* (Belin & Belin, 2000 ▶) have reported that the δ_1*p*_ phase crystallizes in a hexagonal lattice with the space group *P*6_3_/*mmc* (Hahn, 2005 ▶) occupying 52 different crystallographic (Wyckoff) positions. The unit cell is huge including 556 atoms, of which 52 are iron, as shown in Fig. 1 ▶. As can be seen in the [11
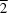
0] projection of the unit cell (Fig. 2 ▶ *a*), two mirror planes exist at *z* = 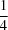 and 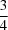 and an inversion centre at the origin. This means that it is sufficient to consider the atomic arrangement only in 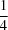 of the unit cell, which is the minimum repeating unit of the [11
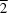
0] projection, to fully describe the crystal structure when viewed along the [11
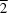
0] direction.^1^ The structure can be considered to consist of a dense packing of zinc-centred polyhedra, which include normal icosahedra [centred at Zn(3), Zn(5), Zn(28), Zn(39), Zn(42) and Zn(44) sites],^2^ bicapped pentagonal prisms [centred at Zn(27) and Zn(45)], 16-atom icosioctahedra [tetracapped truncated octahedra, centred at Zn(4)], and disordered icosahedra [centred at Zn(40)] with positional disorder being at the vertex sites as shown in Fig. 1 ▶. Positional disorder means that the vertex position splits into several different sites with the occupancy for each of the split sites less than unity. However, for a particular vertex position the sum of the occupancies for all the split sites is unity. An icosahedron having such positional disorder is called disordered icosahedra, while the normal icosahedron does not have any positional disorder in the vertex positions. Out of the 52 Fe atoms per unit cell, 32 atoms are located in 2/3 of the vertex sites of disordered icosahedra, while the other 20 atoms are located randomly in mixed sites with zinc throughout the rest of the structure. The vertices of the disordered icosahedron consist of Fe(41), Zn(46), Fe(47), Fe(48), Fe(49), Zn(50), Zn(51) and Fe(52) sites with the occupancies being 1 for Fe(41), 2/3 for Fe(48) and 1/3 for the other six sites. Depending on how positional disorder occurs in the vertex sites of the disordered icosahedron, three different orientations exist for the disordered icosahedron, which are related with each other by 120° rotations about a threefold axis of the hexagonal unit cell at (*x*,*y*) = (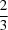, 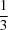).

However, the reported crystal structure of the δ_1*p*_ phase is questionable when referring to the fact that the atomic distance between the Zn(7) and Zn(8) sites is anomalously short (0.74 Å) when compared with the bonding distance in pure zinc (2.79 Å). On top of that, it seems very strange that most Fe atoms are concentrated on disordered icosahedra and that all normal icosahedra are centred by a Zn atom, while the 12 vertex sites are also mostly Zn atoms (actually with mixed occupancies with iron).

## 3. Experimental   

Elements (4N purity) with a molar ratio of Zn:Fe = 97.5:2.5 were sealed in a quartz ampoule under vacuum. The ampoule was heated at 1073 K for 12 h to completely mix the elements. Subsequently, the ampoule was quickly cooled down to 943 K and then slowly cooled down to 808 K over 270 h (−2 K h^−1^), followed by water quenching. We obtained large hexagonal prismatic crystals terminated by pyramidal faces with an approximate size of 8 × 8 × 20 mm by dissolving the zinc matrix with concentrated hydrochloric acid. The crystals were of high quality for single-crystal X-ray diffraction. The chemical composition was measured on several crystals by energy-dispersive X-ray spectroscopy (EDS) in a scanning electron microscope. The average composition was 9.7 ± 0.4 at.%Fe. Some of the crystals were annealed in an evacuated quartz ampoule at 673 K for 168 h followed by furnace-cooling, and we found no change in microstructure and composition, indicating the high thermal stability of the crystal phase. A columnar specimen with the longitudinal axis being parallel to the crystallographic *c* axis was machined from one of the grown single crystals with a Jeol JIB-4000 focused ion-beam (FIB) apparatus at an operating voltage of 30 kV. The crystal size was approximately 28 µm in diameter and 24 µm in length. Synchrotron X-ray diffraction experiments were carried out at 298 K with a large cylindrical image-plate (IP) camera installed at the BL02B1 of SPring-8. The large IP camera enables high statistical data to be obtained. The wavelength of the incident X-ray used was 0.35450 Å (35.00 keV). The crystal structure was solved by direct methods (*SIR97*; Altomare *et al.*, 1999 ▶), and refined by full-matrix least-squares techniques on *F* ^2^ (*SHELXL97*; Sheldrick, 2008 ▶). All calculations were performed with the *WinGX* crystallographic software package (Farrugia, 1999 ▶, 2012 ▶). High-resolution (spatial resolution: ∼ 1.3 Å) scanning transmission electron microscopy (STEM) imaging was made with a Jeol JEM-2100F STEM operated at 200 kV. The probe convergence angle and the inner/outer detector angles for high-angle annular dark-field (HAADF) imaging were 10 and 88–234 mrad, respectively. Ultra-high-resolution (spatial resolution: ≤ 0.8 Å) STEM imaging was made with a Cs-corrected Jeol JEM-ARM200 STEM operated at 200 kV. The probe convergence angle and the inner/outer detector angles for HAADF and annular bright-field (ABF) imaging were 22, 90–370 and 11–22 mrad, respectively. STEM image simulations were performed with the *WinHREM* software package (Ishizuka, 2002 ▶).

## 4. Results   

### 4.1. STEM imaging   

**Figure 3:**
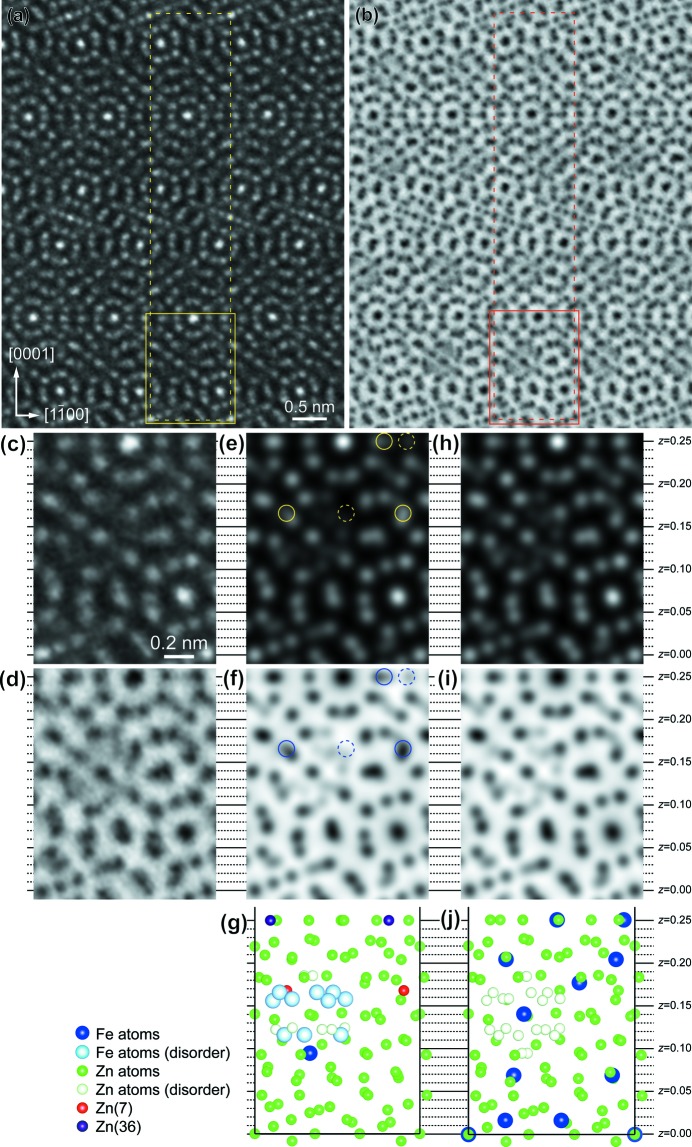
(*a*) HAADF and (*b*) ABF images simultaneously taken along the [11
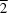
0] zone axis with the Cs-corrected STEM. The framed area corresponds to the projection of the unit cell. (*c*) and (*d*) Magnified images of the parts indicated by a solid frame in (*a*) and (*b*). (*e*) HAADF and (*f*) ABF images calculated with the structural parameters reported by Belin *et al.* (Belin & Belin, 2000 ▶). (*h*) HAADF and (*i*) ABF images calculated with the structural parameters refined in the present study. The corresponding portion of [11
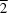
0] projection of the structural models (*g*) reported by Belin *et al.* (Belin & Belin, 2000 ▶) and (*j*) refined in the present study.

Fig. 2 ▶(*b*) indicates an experimental high-resolution STEM HAADF image taken along the [11
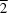
0] zone-axis orientation. The framed area in Fig. 2 ▶(*b*) corresponds to the projection of the unit cell (Fig. 2 ▶ *a*) as reported by Belin *et al.* (Belin & Belin, 2000 ▶). A STEM HAADF image calculated with the atomic coordinates given by Belin *et al.* (Belin & Belin, 2000 ▶) is shown in Fig. 2 ▶(*c*). Some parts of the experimental image of Fig. 2 ▶(*b*) cannot be well reproduced by calculation, as indicated by *A* and *B* in Fig. 2 ▶(*c*). The intensity of bright dots in these areas in the calculated image of Fig. 2 ▶(*c*) is too weak when compared with that in the experimental image of Fig. 2 ▶(*b*). Areas *A* correspond to a part of the disordered icosahedron comprising mostly Fe atoms (dashed circles in Fig. 2 ▶ *a*), while areas *B* correspond to a part of a normal icosahedron centred at Zn(44), according to the result of structure refinement by Belin *et al.* (Belin & Belin, 2000 ▶). However, we cannot deduce the correct atomic coordinates only from the STEM HAADF image of Fig. 2 ▶(*b*) because of the insufficient spatial resolution of ∼ 1.3 Å. We thus employed the Cs-corrected STEM of ultra-high resolution better than 0.8 Å to obtain more detailed information about the correct atomic coordinates. Figs. 3 ▶(*a*) and (*b*) show STEM HAADF and ABF images, respectively, simultaneously taken along the [11
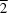
0] zone-axis orientation with the Cs-corrected STEM. The areas framed with a dotted line in Figs. 3 ▶(*a*) and (*b*) correspond to the projection of the unit cell. In the experimental STEM HAADF image of Fig. 3 ▶(*a*), most of the atomic columns are resolved in isolation from other columns in spite of the complex crystal structure. The STEM HAADF and ABF image basically exhibit a reversed image contrast (Findlay *et al.*, 2010 ▶). Figs. 3 ▶(*c*) and (*d*) are magnified images of the areas framed with a solid line (corresponding to 1/4 of the unit cell) in Figs. 3 ▶(*a*) and (*b*), respectively. STEM HAADF and ABF images calculated with the atomic coordinates given by Belin *et al.* (Belin & Belin, 2000 ▶) are shown in Figs. 3 ▶(*e*) and (*f*), respectively. Some inconsistencies between the experimental and calculated images are evidently observed. For both STEM HAADF and ABF images, the intensity of the areas (*z* = ∼ 0.165 and 0.25) indicated by dashed circles in the calculated images is much weaker than that of the corresponding areas in the experimental images. The opposite is true for the areas indicated by open circles in the calculated images. These mean that the *x* and *y* coordinates of the Zn(7) and Zn(36) sites reported by Belin *et al.* (Belin & Belin, 2000 ▶), see Fig. 3 ▶(*g*), are incorrect.

### 4.2. Single-crystal synchrotron X-ray diffraction   

The details of the single-crystal X-ray diffraction are given in Table 1 ▶. The space group *P*6_3_/*mmc* was assigned according to the literature by Belin *et al.* (Belin & Belin, 2000 ▶), and indeed best fitted to our diffraction data. No additional reflections or streaks were observed even when the image plate was overexposed. The structural solution and subsequent refinement yielded 52 crystallographic sites. Table 1 of the supporting information^3^ gives the refined coordinates and isotropic displacement parameters for the δ_1*p*_ phase. In the refinement we employed the following procedures. At first, all atoms were assumed to be Zn with full occupancy because of the small difference in the scattering factors for iron and zinc. Since the eight atomic sites from Zn38 to Zn45 exhibited extraordinarily large isotropic displacement parameters their occupancies were allowed to vary, but all of them eventually converged to be 1/3. Although the occupancies for all the other sites were also allowed to vary after introducing iron, all of them eventually converged to be 1, indicating the absence of positional disorder and vacancies except for the eight atomic sites from Zn38 to Zn45. This means that no sites with mixed occupations of iron and zinc atoms exist in the crystal structure. Fe atoms exclusively occupy the seven atomic sites from Fe1 to Fe7, while Zn atoms exclusively occupy the other sites. The unit cell contains 556 atoms (Pearson symbol *hP*556), of which 52 are Fe atoms and 504 are Zn atoms. The chemical formula for the δ_1*p*_ phase is therefore Fe_13_Zn_126_, instead of FeZn_10_ as previously described by Ghoniem *et al.* and Belin *et al.* (Ghoniem & Lohberg, 1972 ▶; Belin & Belin, 2000 ▶). The composition derived from the structure refinement (9.35 at.%Fe) was in good agreement with that estimated by EDS analysis (9.7 ± 0.4 at.%Fe). Finally, anisotropic displacement parameters for all sites were refined. In Table 1 of the supporting information, the atomic sites are arranged in increasing order of the magnitude of the *c*-axis coordinate for iron and zinc with full occupancy and then zinc with one-third occupancy.

### 4.3. Comparison of X-ray diffraction data with Cs-corrected STEM images   

The Cs-corrected STEM observation has revealed that the atomic coordinates of the two atomic sites [Zn(7) and Zn(36)] reported in the literature by Belin *et al.* (Belin & Belin, 2000 ▶) are incorrect, as shown in Figs. 3 ▶(*c*)–(*g*). Instead, the two sites have been refined as Zn24 and Zn36 sites by single-crystal X-ray diffraction in the present study (see Table 1 of the supporting information). Figs. 3 ▶(*h*) and (*i*) show STEM HAADF and ABF images, respectively, calculated based on the structural model obtained by single-crystal X-ray diffraction in the present study, as schematically illustrated in Fig. 3 ▶(*j*). The calculated images of Figs. 3 ▶(*h*) and (*i*) are in good agreement with the corresponding experimental images (Figs. 3 ▶ *c* and *d*), supporting the validity of the structural model refined in the present study.

## 5. Discussion   

**Figure 4:**
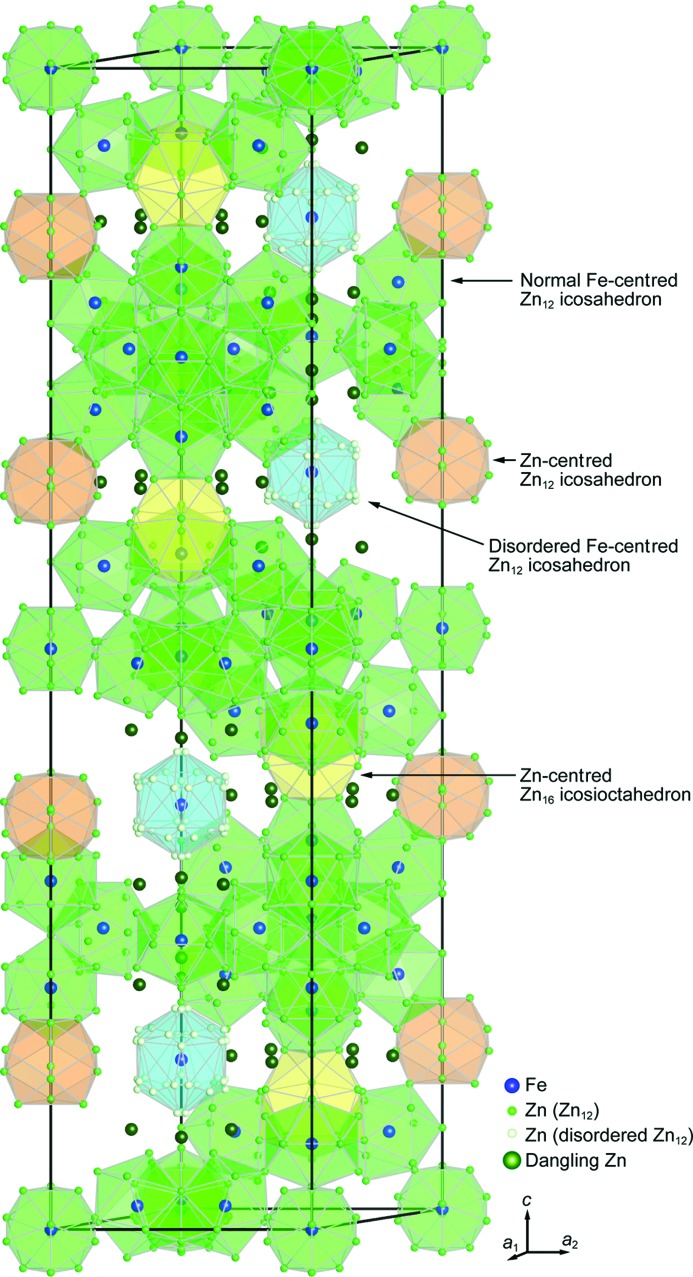
Structural model of the δ_1*p*_ phase refined in the present study, featuring the coordination polyhedra including the iron-centred normal and disordered Zn_12_ icosahedra, the zinc-centred Zn_12_ icosahedra and the Zn_16_ icosioctahedra.

**Figure 5:**
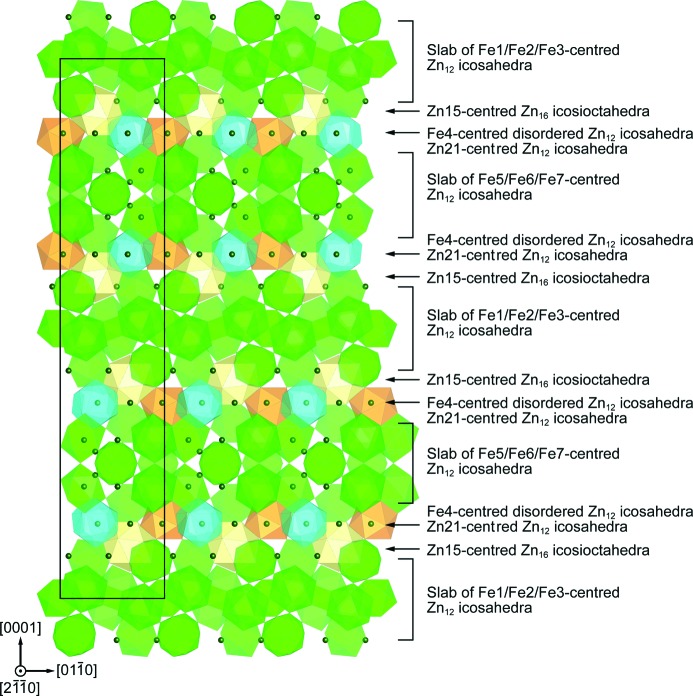
[2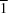 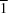
0] projection of the crystal structure of the δ_1*p*_ phase which describes alternate stacking of two types of slabs comprising iron-centred normal Zn_12_ icosahedra (Fe1/Fe2/Fe3-centred and Fe5/Fe6/Fe7-centred). Between the two types of slabs are the iron-centred disordered Zn_12_ icosahedra, zinc-centred icosahedra, zinc-centred icosioctahedra, and dangling Zn atoms that do not constitute any polyhedra. The atoms constituting the vertices of the polyhedra are not depicted in the figure.

The crystal structure of the δ_1*p*_ phase is best understood by considering the packing of coordination polyhedra including normal Zn_12_ icosahedra, disordered Zn_12_ icosahedra and Zn_16_ icosioctahedra, as illustrated in Fig. 4 ▶. All Fe atoms in the seven Wyckoff positions (Fe1–Fe7) exclusively occupy the centre of normal icosahedra (Fe1/Fe2/Fe3 and Fe5/Fe6/Fe7) and disordered icosahedron (Fe4). This is completely different from the structural model reported by Belin *et al.* (Belin & Belin, 2000 ▶), who allocated most Fe atoms to the vertex sites of disordered icosahedra and Zn atoms in the centre of all other constituting polyhedra. In addition to the iron-centred normal and disordered icosahedra, Zn21-centred icosahedra and Zn15-centred icosioctahedra appear in our structural model. The latter also appear in the structural model by Belin *et al.* (Belin & Belin, 2000 ▶; Fig. 1 ▶). The bicapped pentagonal prism (Fig. 1 ▶) described by Belin *et al.* (Belin & Belin, 2000 ▶) does not exist in our structural model. Normal icosahedra, in which Fe1, Fe2 and Fe3 reside at the centre, are connected with each other forming a slab parallel to the *c* plane (Fig. 5 ▶). In the slab, three Fe2-centred icosahedra are connected with one another by face-sharing, whereas Fe1-, Fe2- and Fe3-centred icosahedra are interconnected with each other by sharing Zn atoms at vertices. This is also the case for the Fe5-, Fe6- and Fe7-centred icosahedra. They are connected with each other forming another slab parallel to the *c* plane (Fig. 5 ▶). Three Fe6-centred icosahedra are connected with one another by face-sharing, whereas Fe5-, Fe6- and Fe7-centred icosahedra are interconnected by sharing Zn atoms at vertices. No edge-sharing is observed in the connection among iron-centred icosahedra. The Zn15-centred icosioctahedron is connected with Fe3- and Fe5-centred normal icosahedra by face-sharing so as to bridge two slabs of normal icosahedra. The Zn21-centred icosahedron is connected with Fe6-centred normal icosahedra by face-sharing. On the other hand, the Fe4-centred disordered icosahedron is isolated from all other polyhedra. Out of the 504 Zn atoms, 52 are dangling Zn atoms (Zn13/Zn20/Zn28/Zn34) as indicated by dark grey spheres (dark green in the colour version) in Figs. 4 ▶ and 5 ▶. As the δ_1*p*_ phase can be regarded as a Frank–Kasper phase, the dangling Zn atoms actually reside at the centre of interpenetrating polyhedra. However, it is difficult to accurately define those interpenetrating polyhedra because they consist partly of the partially occupied sites with positional disorder (Zn38–Zn45). The dangling Zn atoms in Zn20 sites are on the same height level as the Fe4-centred disordered icosahedron (with respect to the *c* axis), while dangling Zn atoms in Zn13 sites are located at a level between disordered icosahedra and a slab of normal icosahedra centred by Fe1/Fe2/Fe3 and those in Zn28/Zn34 sites are located at a level between disordered icosahedra and a slab of normal icosahedra centred by Fe5/Fe6/Fe7.

**Figure 6:**
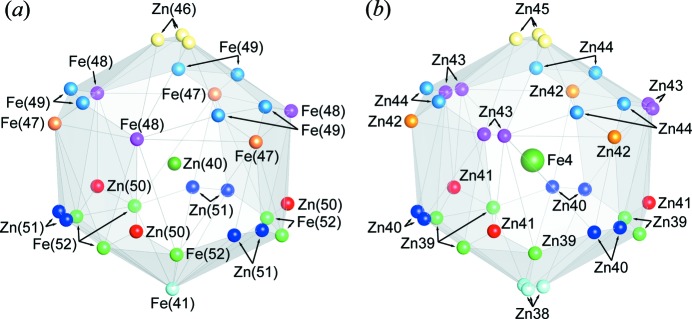
Disordered icosahedron in the structural model (*a*) reported by Belin *et al.* (Belin & Belin, 2000 ▶) and (*b*) refined in the present study.

**Figure 7:**
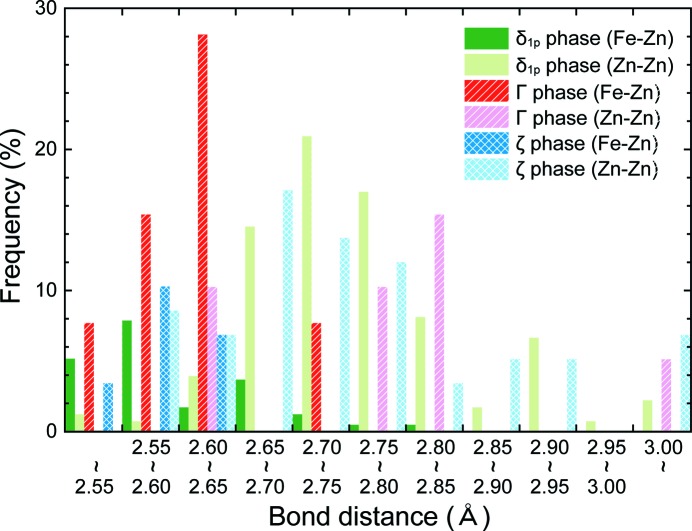
Histogram of the bonding distances for the Fe—Zn and Zn—Zn bonds (shorter than 3.2 Å) in the δ_1p_, Γ and ζ phases (Belin & Belin, 2000 ▶; Belin *et al.*, 2000 ▶).

The disordered icosahedron in our structural model is different from that described in the model by Belin *et al.* (Belin & Belin, 2000 ▶) in two aspects: (i) the occupation behaviour at the centre and vertices, and (ii) positional disorder at the vertices. Belin *et al.* (Belin & Belin, 2000 ▶) have reported that the centre of the disordered icosahedron is occupied by zinc [Zn(40)] and 2/3 of the vertex sites are occupied by Fe atoms (Fig. 6 ▶ *a*). They have further reported that while the occupancy is unity for the bottom vertex site [Fe(41)], those for the Fe(48) site and all other vertex sites with positional disorder are 2/3 and 1/3, respectively. In our structural model, however, the centre of disordered icosahedra is occupied exclusively by iron (Fe4) with all vertices being occupied by Zn atoms (Zn38–Zn45) with an occupancy of 1/3 (Fig. 6 ▶ *b*). The fact that all Fe atoms occupy the centre of normal and disordered Zn_12_ icosahedra indicates that the atomic bonding is stronger for the Fe—Zn bond than for the Zn—Zn bond. This is evident from the shorter average bonding distances for the Fe—Zn bond seen in the histogram of Fig. 7 ▶, in which the bonding distances for the Fe—Zn and Zn—Zn bonds (only for those shorter than 3.2 Å) in the δ_1*p*_ phase are plotted. The same tendency in the bonding characteristics is also observed for the Γ and ζ phases in the iron–zinc system as seen in Fig. 7 ▶ (Belin & Belin, 2000 ▶; Belin *et al.*, 2000 ▶). In fact, there is also a strong tendency for these two phases to form iron-centred Zn_12_ icosahedra. In the Γ phase, four iron-centred Zn_12_ icosahedra are connected with one another in a tetrahedrally close-packed arrangement by face-sharing (Belin & Belin, 2000 ▶), and these tetrahedrally arranged Zn_12_ icosahedra are connected with one another by sharing Zn atoms at vertices. In the ζ phase, on the other hand, iron-centred Zn_12_ icosahedra are linked to one another by vertex-sharing to form a chain of Zn_12_ icosahedra along the *c* axis (Belin *et al.*, 2000 ▶), and dangling Zn atoms are located between the chains as if they act as glue among the chains (Okamoto *et al.*, 2014 ▶). In the δ_1*p*_ phase, however, both types of connection (face- and vertex-sharing) of iron-centred icosahedra and dangling Zn atoms are observed as described above. In view of the fact that the δ_1*p*_ phase exists between the Γ and ζ phases composition-wise in the iron–zinc binary phase diagram (Ghoniem & Lohberg, 1972 ▶; Kubaschewski, 1982 ▶; Nakano *et al.*, 2005 ▶; Kainuma & Ishida, 2005 ▶), the bonding characteristic of the δ_1*p*_ phase also seems to be intermediate. One point to be noted here is that there is a common characteristic for all these intermetallic phases in the iron–zinc system that Zn_12_ icosahedra are usually formed with an Fe atom occupying the centre, most probably due to the fact that the atomic bonding is stronger for the Fe—Zn bond than for the Zn—Zn bond. From this point of view, it seems very unfavourable for Fe atoms to occupy 2/3 the vertex sites of a zinc-centred disordered icosahedron, as reported by Belin *et al.* (Belin & Belin, 2000 ▶).

## 6. Conclusions   

The crystal structure of the δ_1*p*_ phase in the iron–zinc system has been refined by single-crystal synchrotron X-ray diffraction combined with Cs-corrected STEM. The large hexagonal unit cell of the δ_1*p*_ phase with the space group of *P*6_3_/*mmc* is best described by considering the packing of coordination polyhedra including normal Zn_12_ icosahedra, disordered Zn_12_ icosahedra and Zn_16_ icosioctahedra. All Fe atoms exclusively occupy the centre of normal and disordered icosahedra. Normal icosahedra constitute two types of slabs stacked alternately along the *c*-axis, being bridged with each other by face-sharing with icosioctahedra. Disordered icosahedra are isolated from all other polyhedra. The unit cell contains 556 atoms (Pearson symbol *hP*556), of which 52 are Fe atoms and 504 are Zn atoms so that the chemical formula for the δ_1*p*_ phase is expressed as Fe_13_Zn_126_ rather than FeZn_10_. The connecting features of the iron-centred Zn_12_ icosahedra in the δ_1*p*_ phase are intermediate between those in the Γ and ζ phases. It is the high statistical precision stemming from the X-ray source of high luminance as well as the image plate of a wide dynamic range that makes it possible to distinguish iron from zinc in the present study. Cs-corrected ultra-high-resolution STEM imaging is also powerful to directly detect discrepancies between the real structure and structural models through direct observations of individual atomic columns even when the crystal structure is considerably complicated.

## Supplementary Material

Crystal structure: contains datablock(s) I. DOI: 10.1107/S2052520613034410/dk5020sup1.cif

Structure factors: contains datablock(s) delta1p. DOI: 10.1107/S2052520613034410/dk5020Isup2.hkl

Table of atomic coordinates. DOI: 10.1107/S2052520613034410/dk5020sup3.pdf

CCDC reference: 978356

## Figures and Tables

**Table 1:** Summary of crystallographic data and structure refinement for δ_1*p*_

Crystal data	
Chemical formula	Fe_13_Zn_126_
*M*_r_	8963.89
Crystal system, space group	Hexagonal, *P*6_3_/*m* *m* *c*
Temperature (K)	300
*a*, *c* (Å)	12.8297 (2), 57.286 (1)
*V* (Å^3^)	8166.0 (3)
*Z*	4
Radiation type	Synchrotron, λ = 0.35450 Å
μ (mm^−1^)	20.094
Crystal size (mm)	0.028 × 0.028 × 0.024
	
Data collection	
Diffractometer	HUBER 4-circle
Absorption correction	–
No. of measured, independent and observed [*I* > 2σ(*I*)] reflections	203 263, 16 996, 14 411
*R*_int_	0.028
(sin θ/λ)_max_ (Å^−1^)	1.111
Completeness to θ = 23.19°	99.9%

Refinement	
*R*[*F* ^2^ > 2σ(*F* ^2^)], *wR*(*F* ^2^), *S*	0.031, 0.127, 1.03
No. of reflections	16 996
No. of parameters	308
Δρ_max_, Δρ_min_ (e Å^−3^)	5.91, −4.16

Computer programs: *CrystalClear* (Rigaku, 2006 ▶), *SORTAV* (Blessing, 1995 ▶), *SIR92* (Altomare *et al.*, 1999 ▶), *SHELXL97* (Sheldrick, 2008 ▶), *WinGX* publication routines (Farrugia, 1999 ▶).

## Footnotes

1 The asymmetric unit of the space group *P*6_3_/*mmc* has a volume of 1/12 of the unit cell.

2 Atom labels with and without parentheses correspond to those reported by Belin *et al.* (Belin & Belin, 2000 ▶) and determined in the present study, respectively.

3 Supporting information for this paper is available from the IUCr electronic archives (Reference: DK5020).

## References

[bb1] Allen, C. & Mackowiak, J. (1963). *Corros. Sci.* **3**, 87–97.

[bb2] Altomare, A., Burla, M. C., Camalli, M., Cascarano, G. L., Giacovazzo, C., Guagliardi, A., Moliterni, A. G. G., Polidori, G. & Spagna, R. (1999). *J. Appl. Cryst.* **32**, 115–119.

[bb3] Bastin, G. F., Vanloo, F. J. J. & Rieck, G. D. (1974). *Z. Metallkdd.* **65**, 656–660.

[bb4] Bastin, G. F., Vanloo, F. J. J. & Rieck, G. D. (1977). *Z. Metallkdd.* **68**, 359–361.

[bb5] Belin, C. H. E. & Belin, R. C. H. (2000). *J. Solid State Chem.* **151**, 85–95.

[bb6] Belin, R., Tillard, M. & Monconduit, L. (2000). *Acta Cryst.* C**56**, 267–268. 10.1107/S0108270199014997 10777914

[bb32] Blessing, R. H. (1995). *Acta Cryst.* A**51**, 33–38. 10.1107/s0108767394005726 7702794

[bb7] Brandon, J. K., Brizard, R. Y., Chieh, P. C., McMillan, R. K. & Pearson, W. B. (1974). *Acta Cryst.* B**30**, 1412–1417.

[bb8] Brown, P. J. (1962). *Acta Cryst.* **15**, 608–612.

[bb9] Farrugia, L. J. (1999). *J. Appl. Cryst.* **32**, 837–838.

[bb10] Farrugia, L. J. (2012). *J. Appl. Cryst.* **45**, 849–854.

[bb11] Findlay, S. D., Shibata, N., Sawada, H., Okunishi, E., Kondo, Y. & Ikuhara, Y. (2010). *Ultramicroscopy*, **110**, 903–923. 10.1016/j.ultramic.2010.04.004 20434265

[bb12] Ghoniem, M. A. & Lohberg, K. (1972). *Metall*, **26**, 1026.

[bb13] Hahn, T. (2005). *International Tables for Crystallography*, Vol. A, *Space-Group Symmetry*, 5th ed. Dordrecht, The Netherlands: Springer.

[bb14] Hong, M. H. & Saka, H. (1997). *Scr. Mater.* **36**, 1423–1429.

[bb15] Ishizuka, K. (2002). *Ultramicroscopy*, **90**, 71–83. 10.1016/s0304-3991(01)00145-0 11942640

[bb16] Johansson, A., Ljung, H. & Westman, S. (1968). *Acta Chem. Scand.* **22**, 2743–2753.

[bb17] Kainuma, R. & Ishida, K. (2005). *Tetsu Hagane*, **91**, 349–355.

[bb18] Kato, C., Koumura, H., Uesugi, Y. & Mochizuki, K. (1994). *TMS Annual Meeting, The Physical Metallurgy of Zinc Coated Steel*, edited by A. R. Marder, pp. 241–249. San Francisco, CA: TMS.

[bb19] Koster, A. S. & Schoone, J. C. (1981). *Acta Cryst.* B**37**, 1905–1907.

[bb20] Kubaschewski, O. (1982). *Iron-Binary Phase Diagrams.* Berlin: Springer-Verlag.

[bb21] Marder, A. R. (2000). *Prog. Mater. Sci.* **45**, 191–271.

[bb22] Nakano, J., Malakhov, D. V. & Purdy, G. R. (2005). *Calphad*, **29**, 276–288.

[bb23] Okamoto, N. L., Kashioka, D., Inomoto, M., Inui, H., Takebayashi, H. & Yamaguchi, S. (2013). *Scr. Mater.* **69**, 307–310.

[bb24] Okamoto, N. L., Inomoto, M., Adachi, H., Takebayashi, H. & Inui, H. (2014). *Acta Mater.* **65**, 229–239.

[bb25] Perrot, P. & Dauphin, J. (1988). *Calphad*, **12**, 33–40.

[bb26] Petersen, S., Spencer, P. J. & Hack, K. (1988). *Thermochim. Acta*, **129**, 77–87.

[bb27] Reumont, G., Perrot, P., Fiorani, J. M. & Hertz, J. (2000). *J. Phase Equilib.* **21**, 371–378.

[bb31] Rigaku (2006). *CrystalClear* Rigaku Corporation, Tokyo, Japan.

[bb28] Sheldrick, G. M. (2008). *Acta Cryst.* A**64**, 112–122. 10.1107/S0108767307043930 18156677

[bb29] Su, X., Tang, N. & Toguri, J. M. (2001). *J. Alloys Compd.* **325**, 129–136.

[bb30] Uwakweh, O. N. C., Liu, Z., Jordan, A., Chakoumakos, B., Spooner, S. & Maziasz, P. (2000). *Metall. Mater. Trans. A*, **31**, 2739–2745.

