# Supplementary material for: Structure refinement of the δ1p phase in the Fe–Zn system by single-crystal X-ray diffraction combined with scanning transmission electron microscopy
Source: Acta Crystallogr B Struct Sci Cryst Eng Mater. 2014 Mar 17;70(Pt 2):275–82. doi: 10.1107/S2052520613034410 (PMC3970753; doi:10.1107/S2052520613034410)
Supplement: Supplementary file 3 [file b-70-00275-sup3.pdf]

Acta Crystallographica Section B

Volume 70 (2014)

Supporting information for article:

Structure refinement of the δ1*p* phase in the Fe–Zn system by single-crystal X-ray diffraction combined with scanning transmission electron microscopy

Norihiko L. Okamoto, Katsushi Tanaka, Akira Yasuhara and Haruyuki Inui

Table 1 Atomic coordinates and equivalent isotropic displacement parameters for δ_{1p}. U_{eq} is defined as one-third of the trace of the orthogonalized U^{ij} tensor.

Atom	Wyckoff	Occ.	*x*	*y*	*z*	U_{eq} (Å^2)
Fe1†	2*a*	1	0	0	0	0.005(1)
Fe2†	12*k*	1	0.7790(1)	0.5579(1)	0.0164(1)	0.008(1)
Fe3†	12*k*	1	0.1360(1)	0.2720(1)	0.0689(1)	0.007(1)
Fe4‡	4*f*	1	2/3	1/3	0.1403(1)	0.007(1)
Fe5†	4*f*	1	1/3	2/3	0.1768(1)	0.006(1)
Fe6†	12*k*	1	0.1129(1)	0.2258(1)	0.2040(1)	0.008(1)
Fe7†	6*h*	1	0.4671(1)	0.9342(1)	1/4	0.006(1)
Zn1	12*i*	1	0.3626(1)	0	0	0.013(1)
Zn2	12*k*	1	0.8870(1)	0.7739(1)	0.0083(1)	0.010(1)
Zn3	12*k*	1	0.4098(1)	0.8196(1)	0.0132(1)	0.018(1)
Zn4	4*f*	1	2/3	1/3	0.0243(1)	0.014(1)
Zn5	12*k*	1	0.1950(1)	0.3900(1)	0.0277(1)	0.014(1)
Zn6	12*k*	1	0.5473(1)	0.0946(1)	0.0313(1)	0.016(1)
Zn7	12*k*	1	0.0707(1)	0.1414(1)	0.0346(1)	0.010(1)
Zn8	24*l*	1	0.3146(1)	0.0440(1)	0.0451(1)	0.014(1)
Zn9	4*f*	1	1/3	2/3	0.0490(1)	0.014(1)
Zn10	12*k*	1	0.7489(1)	0.4978(1)	0.0614(1)	0.020(1)
Zn11	24*l*	1	0.1293(1)	0.4683(1)	0.0652(1)	0.018(1)
Zn12	12*k*	1	0.9264(1)	0.8528(1)	0.0725(1)	0.014(1)
Zn13$^{\#}$	12*k*	1	0.5390(1)	0.0780(1)	0.0780(1)	0.017(1)
Zn14	24*l*	1	0.3177(1)	0.0390(1)	0.0925(1)	0.019(1)
Zn15¶	4*f*	1	1/3	2/3	0.0957(1)	0.020(1)
Zn16	12*k*	1	0.2035(1)	0.4069(1)	0.1046(1)	0.020(1)
Zn17	12*k*	1	0.0834(1)	0.1668(1)	0.1093(1)	0.022(1)
Zn18	12*k*	1	0.4506(1)	0.9012(1)	0.1107(1)	0.016(1)
Zn19	12*k*	1	0.8808(1)	0.7615(1)	0.1339(1)	0.023(1)
Zn20$^{\#}$	24*l*	1	0.0247(1)	0.3559(1)	0.1378(1)	0.021(1)
Zn21†	4*e*	1	0	0	0.1406(1)	0.012(1)
Zn22	12*k*	1	0.2632(1)	0.5263(1)	0.1414(1)	0.021(1)
Zn23	12*k*	1	0.1156(1)	0.2312(1)	0.1569(1)	0.017(1)
Zn24	12*k*	1	0.4508(1)	0.9016(1)	0.1678(1)	0.017(1)
Zn25	12*k*	1	0.9295(1)	0.8591(1)	0.1804(1)	0.016(1)
Zn26	24*l*	1	0.3235(1)	0.0248(1)	0.1832(1)	0.019(1)
Zn27	12*k*	1	0.2179(1)	0.4358(1)	0.1854(1)	0.016(1)
Zn28$^{\#}$	12*k*	1	0.7737(1)	0.5473(1)	0.2068(1)	0.020(1)
Zn29	12*k*	1	0.5308(1)	0.0616(1)	0.2095(1)	0.016(1)

Zn30	12*k*	1	0.4062(1)	0.8125(1)	0.2116(1)	0.013(1)
Zn31	4*e*	1	0	0	0.2198(1)	0.014(1)
Zn32	12*k*	1	0.8832(1)	0.7665(1)	0.2242(1)	0.022(1)
Zn33	24*l*	1	0.0626(1)	0.3612(1)	0.2264(1)	0.015(1)
Zn34[#]	4*f*	1	2/3	1/3	0.2281(1)	0.017(1)
Zn35	6*h*	1	0.2617(1)	0.5234(1)	1/4	0.014(1)
Zn36	6*h*	1	0.0954(1)	0.1908(1)	1/4	0.014(1)
Zn37	12*j*	1	0.4588(1)	0.1319(1)	1/4	0.019(1)
Zn38	12*k*	1/3	0.6773(1)	0.3546(1)	0.0947(1)	0.018(1)
Zn39	24*l*	1/3	0.4821(1)	0.1819(1)	0.1153(1)	0.028(1)
Zn40	24*l*	1/3	0.5370(1)	0.1280(1)	0.1220(1)	0.021(1)
Zn41	12*k*	1/3	0.7742(1)	0.5485(1)	0.1234(1)	0.026(1)
Zn42	12*k*	1/3	0.5546(1)	0.1092(1)	0.1560(1)	0.025(1)
Zn43	24*l*	1/3	0.4496(1)	0.2079(1)	0.1582(1)	0.019(1)
Zn44	24*l*	1/3	0.5251(1)	0.1485(1)	0.1652(1)	0.032(1)
Zn45	12*k*	1/3	0.6508(1)	0.3016(1)	0.1842(1)	0.019(1)

[†]Centre of the normal Zn_{12} icosahedra

[‡]Centre of the disordered Zn_{12} icosahedron

[¶]Centre of the Zn_{16} icosioctahedron

[#]Dangling zinc atoms
